# Qualitative Characterization of Unrefined Durum Wheat Air-Classified Fractions

**DOI:** 10.3390/foods10112817

**Published:** 2021-11-16

**Authors:** Alessandro Cammerata, Barbara Laddomada, Francesco Milano, Francesco Camerlengo, Marco Bonarrigo, Stefania Masci, Francesco Sestili

**Affiliations:** 1 Council for Agricultural Research and Economics, Research Centre for Engineering and Agro-Food Processing, Via Manziana 30, 00189 Rome, Italy; alessandro.cammerata@crea.gov.it; 2 Institute of Sciences of Food Production (ISPA), National Research Council (CNR), Via Monteroni, 73100 Lecce, Italy; f.camerlengo@unitus.it (F.C.); bonarrigo.marco@yahoo.it (M.B.); masci@unitus.it (S.M.); 3 Department of Agriculture and Forest Sciences (DAFNE), University of Tuscia, Via San Camillo de Lellis snc, 01100 Viterbo, Italy; francesco.milano@cnr.it

**Keywords:** durum wheat, air-classified fractions, alveographic properties, antioxidants, starch, ATI

## Abstract

Durum wheat milling is a key process step to improve the quality and safety of final products. The aim of this study was to characterize three bran-enriched milling fractions (i.e., F250, G230 and G250), obtained from three durum wheat grain samples, by using an innovative micronization and air-classification technology. Milling fractions were characterized for main standard quality parameters and for alveographic properties, starch composition and content, phenolic acids, antioxidant activity and ATIs. Results showed that yield recovery, ash content and particle size distributions were influenced either by the operating conditions (230 or 250) or by the grain samples. While total starch content was lower in the micronized sample and air-classified fractions, the P/L ratio increased in air-classified fractions as compared to semolina. Six main individual phenolic acids were identified through HPLC-DAD analysis (i.e., ferulic acid, vanillic acid, *p*-coumaric acid, sinapic acid, syringic and *p*-hydroxybenzoic acids). Compared to semolina, higher contents of all individual phenolic components were found in all bran-enriched fractions. The highest rise of TPAs occurred in the F250 fraction, which was maintained in the derived pasta. Moreover, bran-enriched fractions showed significant reductions of ATIs content versus semolina. Overall, our data suggest the potential health benefits of F250, G230 and G250 and support their use to make durum-based foods.

## 1. Introduction

Durum wheat (*Triticum turgidum* L. ssp. *durum*) is an important crop, especially in the Mediterranean basin, as it is used for the production of daily foods, such as pasta, couscous, bulgur, and unleavened and leavened bread. The main component of the kernel is starch, followed by storage proteins, lipids and other minor compounds. Durum wheat grain also contains several bioactive compounds of health interest, such as insoluble fiber, phenolic acids, and alkylresorcinols, which are mostly concentrated within the coating structure of the kernel [1]. Phenolic acids are among the most abundant and studied components promoting human health. As dietary antioxidants, they act as free-radical scavengers [2,3], and reduce the inflammatory response in endothelial cells and monocytes [4]. Besides their antioxidant and antiradical activity, phenolic acids participate in plant cell walls as structural components and are involved in plant adaptation to abiotic and biotic stresses [5,6]. Among other wheat constituents that recently have become a major target of research in wheat there are the Amylase/Trypsin Inhibitors (ATIs) [7]. ATIs contribute about 3% of total kernel proteins and, similarly to phenolic acids, they are involved in plant defense mechanisms against pests and pathogens [7]. However, differently from them, ATI proteins are concentrated mainly in the endosperm and are associated with negative effects on human health [7]. ATIs, in fact, are putative factors triggering Non-Celiac Wheat Sensitivity (NCWS), one of the most common adverse reactions to wheat, and are responsible for baker’s asthma, the most common occupational Ig-E mediated allergy in Europe [7].

So far, the use of raw materials rich in health-promoting compounds and poor in toxic components constitutes a key point for a proper nutritional approach [8,9,10]. Diets rich in whole cereals, legumes, vegetables and fruit guarantee greater protection against the onset of age-related metabolic diseases that are mainly spread in developed countries [11,12]. Compared to refined products, whole grain-based foods, besides having higher contents of dietary fiber and bioactive compounds, are characterized by reduced amounts of starch, which is associated with postprandial responses, the risk of type 2 diabetes and the incidence of other non-communicable diseases, such as cardiovascular disorders and colon cancer [13,14,15].

Several attempts were made to improve the poor quality of refined wheat milling products. Among these, the technology based on micronization and subsequent air-classification of milling particles was among the most promising [1,16]. The air-classification technology is a suitable tool to this purpose giving rise to sub-fractions with diverse particle size and composition that can be selected to enhance the beneficial health effects associated with bran particles, while limiting the content of toxic contaminants [17,18,19,20,21]. Through the use of this latter technology, several types of unrefined milling products can be directly obtained from the air-classified plants and evaluated on the basis of qualitative tests without the need for adding selected fractions enriched with specific outer layers such as aleurone [22,23]. Air-classification is based on pneumatic transportation of micronized samples inside the plant, where a series of ascending airflows are controlled by setting the airflow inlet valve at different opening conditions [17]. At the end of each cycle, G fractions (heavier gross particles) and F fractions (fine particles), are collected. The use of micronization and subsequent air-classification treatment has expanded the possibility of selecting milling fractions with technological and nutritional characteristics that are more suitable to make healthier and safer unrefined wheat-based products, also taking into account good testing results and consumer acceptability [16]. These latter aspects are of great interest because durum end-products (e.g., pasta, bread) must meet good technological requirements and consumers’ taste. Moreover, constant upgrading of micronization and air-classification technologies significantly improved their efficiency over the last decade. Among the main innovative solutions, a Programmable Logic Control (PLC) used to manage the airflow inside the separation chamber and a decreasing section chamber were adopted [17]. The latter modifications allowed to better split heavier gross fractions (G) from fine fractions (F). Three air-classified fractions of major interest resulted, namely F250, G230 and G250, showing a good compromise between technological properties and bran enrichment [17].

More in detail, these three fractions proved to be the most interesting on the basis of the following criteria:

- F250 (unrefined product with higher bran content): excellent yield (60–70%), good particle size composition, higher ash percentage than micronized samples, and mycotoxin presence;

- G250 (unrefined product with more semolina particles and less fine middlings): insufficient yield (20–30%) but excellent particle size composition, good ash content (higher than semolina), and strong reduction of the mycotoxin content;

- G230 (unrefined product containing both more semolina and more fine middlings): excellent yield (>60%), balanced particle size composition, good ash content (higher than semolina), and strong reduction of the mycotoxin content.

In the present work, the air-classified fractions F250, G230 and G250 were thoroughly characterized in terms of main quality parameters, alveographic properties, starch composition, phenolic acids profile and antioxidant activity, along with the measure of ATIs amount.

## 2. Materials and Methods

### 2.1. Plant Materials

Three grain samples were considered in the present study, each belonging to: (i) cv. Saragolla, grown in the Lazio region (hereafter named: Saragolla_LA); (ii) cv. Antalis, grown in the Basilicata region (hereafter named: Antalis_BA); and (iii) cv. Antalis, grown in the Marche region (hereafter named: Antalis_MA). The plants were grown in experimental fields under conventional farming during the 2018–2019 growing season.

### 2.2. Grain Characterization

**Figure 1:**
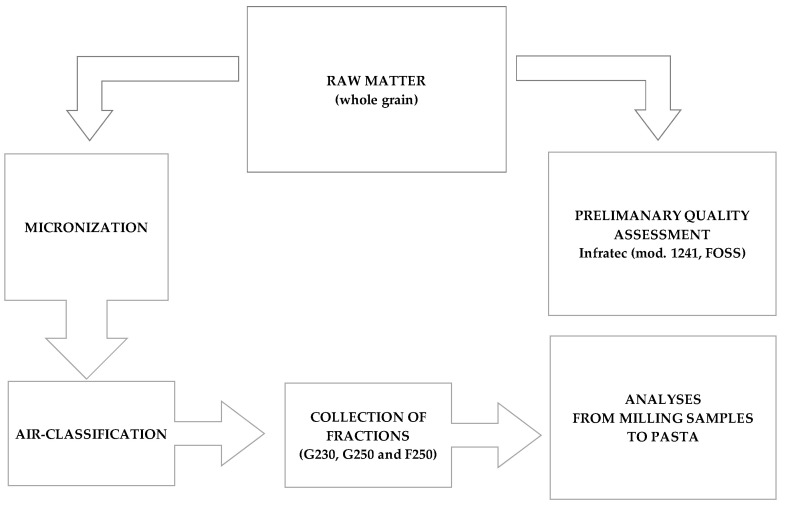
Flow chart of the experimental plan.

Grain samples were evaluated for test weight, moisture and protein content, gluten percentage and yellow color through near-infrared analysis in transmission mode (NIT) by using *Infratec*™ mod 1241 (FOSS, Hillerød, Denmark). Figure 1 shows the flow chart of the experimental plan.

### 2.3. Whole Grain Micronizationand Air Classification

Durum wheat grain samples (11 kg per each grain sample) were micronized using a micronizer pilot plan (mod. 32300, KMXi-300-7,5; Separ Microsystem S.a.s, Brescia, Italia). The micronization step did not require a preventive conditioning of the grains. The micronizing pilot plant was equipped with a hammer crusher impeller with a reduced cross-section inside the grinding chamber. Compared to the traditional type, a sieving grid (Ø = 0.7 mm) suitable to obtain a more homogeneous product was added to the plan. Afterwards, the micronized sample was submitted to an air-classifier pilot plant (model SX-LAB; Separ Microsystem S.a.s, Brescia, Italia) suitable for a particle size up to a setting limit (Ø ≤ 1.5 mm). More in detail, micronized aliquots of 2.0 kg were air-classified for each cycle (in total 3–5 cycles) at a time by setting the airflow inlet valve at 230 and 250. At the end of each cycle the fractions of type G (heavier gross particles) and F (fine particles) were collected but only G230, G250 and F250 were submitted to analysis. All steps of the milling process were carried out both using the plants already updated and following the detailed procedure recently described [17].

### 2.4. Traditional Roller Milling Process

Grain samples were conditioned by adding water until a moisture value of 17% was achieved and left to rest for 24 h. Such a specific treatment was helpful to favor both the undressing process of kernels and softening the endosperm, thus being suitable to submit grain samples to the traditional roller milling plant (Bühler, model MLU 202, Uzwil, Switzerland). The main milling products collected were semolina refined through a sieving treatment (sieve types: 38GG, 40GG and 44GG) by the use of a suitable pilot plant sieving system (NAMAD Impianti, Rome, Italy), bran fractions (coarse and refined types) and fine middlings.

### 2.5. Yield, Ash and Particle Size

Mean yield percentages of the milling fractions were calculated as the weight percentage over the weight of the starting sample. Ash content was determined by following the official method EN ISO 2171:2010 [24]. Particle size analysis of each fraction was carried out using certified test sieves (Giuliani Tecnologie S.r.l., Turin, Italy). Concerning this last point, an electronic sieve (Giuliani Tecnologie S.r.l., Turin, Italy, mod. IG/3) consisting of six stacked sieves of different screens (500-425-355-250-180-125 µm) was used. The milling fraction retained in each sieve was then weighed. The yield parameter was used as a key indicator of the milling quality along with ash and grain size parameters; these last two are expressly mentioned in the Italian legislation [25].

### 2.6. Alveographic Parameters

The alveographic parameters were evaluated on the basis of the viscoelastic behavior of the dough. The analyses were performed using the *Chopin* alveograph (model NG) AACC Method 54-30.02 and UNI 10453 (Cedex, France) [26].

### 2.7. Total Starch, Damaged Starch and Amylose

Starch content and damaged starch were determined using the Total Starch Assay Kit (K-TSTA, Megazyme, Irishtown, Ireland) and the Starch Damage Assay Kit (K-SDAM, Megazyme, Irishtown, Ireland), respectively. Total starch contents were determined using the protocol specific for “samples containing also resistant starch”. For each fraction, the values represented the mean of four technical replicates.

Amylose content was determined from 15 mg of sample using a colorimetric assay based on the iodine–amylose reaction [27]. The standard curve was created using a mixture of potato amylose (Fluka, Neu-Ulm, Germany) and wheat amylopectin (Sigma Aldrich, St. Louis, MO, USA). Each value represented the mean of four technical replicates.

### 2.8. Phenolic Acids Analysis

Phenolic acid analysis was performed on the raw materials (micronized samples; F250, G250 and G230 air classification fractions; and semolina) and on uncooked and cooked pasta made using the F250, G250 and G230 air classification fractions and semolina as a control. Total phenolic acids (TPA), comprising the soluble and insoluble fractions, were extracted from samples and analyzed by HPLC according to the procedure detailed in Laddomada et al. [28]. In brief, samples underwent delipidation using hexane, hydrolyzation with 2M NaOH, and acidification with HCl 12 M up to pH 2 prior to ethyl acetate extraction. Ethyl acetate extracts were dried under nitrogen flux, dissolved in 80:20 methanol/water and analyzed using an Agilent 1100 Series HPLC-DAD system (Agilent Technologies, Santa Clara, CA, USA). Individual phenolic acids were identified by comparing their retention times and UV–Vis spectra to those of authentic phenolic standards and quantified via their ratio to the internal standard (3, 5-dichloro-4-hydroxybenzoic acid) added to every sample and using calibration curves for this standard. All analyses were performed on duplicate extracts.

### 2.9. Pasta making Process

Semolina (control) and the F250, G250 and G230 air-classified fractions (1 kg) were mixed with 280 mL water in a premixing chamber for 15 min; afterwards the dough was transferred to a pilot plan extruder (NAMAD impianti, Rome, Italy) equipped with a 1.6 mm Teflon-coated spaghetti die. Fresh spaghetti were dried using a pilot plan drier (AFREM, Lyon, France), at 50° C for 18 h. Dried pasta samples were stored in sealed plastic bags at room temperature.

### 2.10. Antioxidant Activity Assayes

Trolox equivalent antioxidant capacity (TEAC) was measured for all extracts using the ABTS decolorization assay according to Durante et al. [29] with modifications. ABTS^+^ stock solution was prepared by incubating overnight in the dark 7 mM ABTS (2,2′-azinobis (3-ethylbenzothiazoline-6-sulfonic acid) and 2.45 mM potassium persulfate in water. Trolox standard solutions in the interval of 0–300 µM were prepared by diluting in 80:20 methanol/water the ethanolic 30 mM stock solution. Samples were diluted in 80:20 methanol/water by a factor varying between 10 and 80 according to their TPAcontent and mixed with diluted ABTS^+^ (A_734_ = 0.7) solution in PBS (Phosphate Buffer Solution) (50 µL samples in duplicate or Trolox standard in 950 µL ABTS^+^). After 5 min of incubation at 25 °C, the absorbance at 734 nm was measured by means of a spectrometer (Shimadzu UV-1800). TEAC values were calculated from the Trolox standard curve and values in µeq/mL were converted into µeq/g dry matter considering the initial amount of samples used for the TPA extraction.

### 2.11. Extraction of Albumin and Globulin Fractions (A/G)

The A/G fraction was extracted according to Lupi et al. [30]. Briefly, 1 g of wheat semolina or air-classified fractions were suspended in 27 mL of 0.05 M phosphate buffer/0.1 M NaCl (pH = 7.8) and incubated for 2 h at 4 °C. After centrifugation at 8000× *g* for 15 min at 15 °C, the supernatant was recovered, and the proteins (A/G fraction) were precipitated with four volumes of cold (−20 °C) acetone. After 1 h, the supernatant was discarded and the protein pellet was dissolved in 50 mM carbonate buffer (pH = 9.6).

### 2.12. Indirect Enzyme-Linked Immunosorbent Assay (ELISA) with Anti-ATI Antibodies

The wells of a microtiter plate (ELISA plate 82.1582.100, Sarstedt, Nümbrecht, Germany) were coated with 5 µg/mL of antigen prepared as above in 50 mM carbonate buffer (pH = 9.6) overnight at 4 °C. The plate was washed three times with PBS-0.05% Tween 20, and then the wells were blocked with PBS-BSA 4% for 1 h at 37 °C. After three washes with PBS-0.05% Tween 20, the plate was incubated for 1 h at 37 °C with serial dilutions (from 1:2 to 1:20,000) in PBS-BSA 2% of anti-ATI polyclonal antibodies (developed by BIA-INRA, Nantes, France). Following three washes with PBS-0.05% Tween 20, the secondary antibody (anti-rabbit IgG conjugated with horseradish peroxidase) diluted 1:3000 in PBS-BSA 2% was added to each well and the plate was incubated for 1 h at 37 °C. After three additional washings with PBS-0.05% Tween 20, the plate was incubated at room temperature with the colorimetric substrate, composed of o-phenylenediamine (OPD) in 0.05 M citrate buffer (pH = 5.5) plus hydrogen peroxide. After 30 min, the reaction was stopped with H_2_SO_4_ 4N and the plate was read at 492 nm (Multiskan GO, Thermo Scientific). Three technical replicates were used and the data were subjected to one-way ANOVA followed by Tukey’s post hoc test. When one outlier was present, this was excluded from analysis.

### 2.13. Statistical Analysis

With regards to all data the analysis of variance was performed applying ANOVA (post-hoc: Tukey test) by using the statistical software PAST 2.12 [31].

## 3. Results

### 3.1. Grain Characterization

The main qualitative parameters of grain samples used are summarized in Table 1. Test weight values ranged from 81.0 to 84.4 kg/hl, revealing the absence of significant quantities of shriveled kernels, thus falling in the first-class group according to the official standards UNI 10709:1998 [32]. The moisture content of the grains varied from 10.2% to 10.3%, therefore falling below the maximum limit of 14.0%. Except for a low yellow color, protein and gluten percentages were satisfactory, varying from 12.7% to 15.0% (protein content), and from 8.7% to 10.5% (gluten).

### 3.2. Milling Yield and Particle Size Parameters

**Figure 2:**
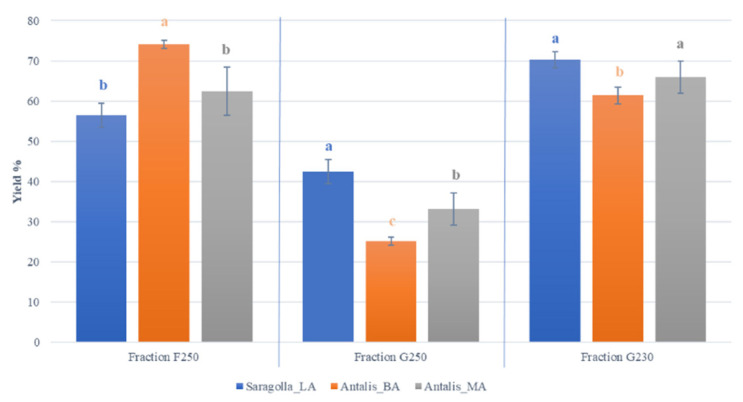
Mean yield (%) of F250, G250 and G230 air-classified fractions obtained from Saragolla_LA, Antalis_BA and Antalis_MA samples. Different letters indicate statistically significant differences (*p* < 0.05, *n* = 3) within each fraction type. F250: Saragolla_LA = 3.4 kg, Antalis_BA = 4.5 kg, Antalis_MA = 3.9 kg; G250: Saragolla_LA = 2.5 kg, Antalis_BA = 1.5 kg, Antalis_MA = 1.9 kg; G230: Saragolla_LA = 3.5 kg, Antalis_BA = 3.0 kg, Antalis_MA = 3.3 kg.

The average yield recovery of micronized samples exceeded 99%, thus showing a negligible loss of the starting grain samples. Yield percentages of air-classified fractions were high for the F250 and G230 milling fractions (Figure 2). Some significant differences were observed within each F and G fraction as depending on grain samples. In fact, within G milling products, Saragolla_LA yields varied from 42% (G250 setting) to 70% (G230 setting), whereas Antalis_BA yield recovery was lower and comprised between 25% (G250) and 61% (G230), and that of Antalis_MA ranged from 33% (G250) to 66% (G230). Among F fractions, yields varied from a minimum of 56% (Saragolla_LA) to a maximum of 74% (Antalis_BA) (Figure 2).

**Figure 3:**
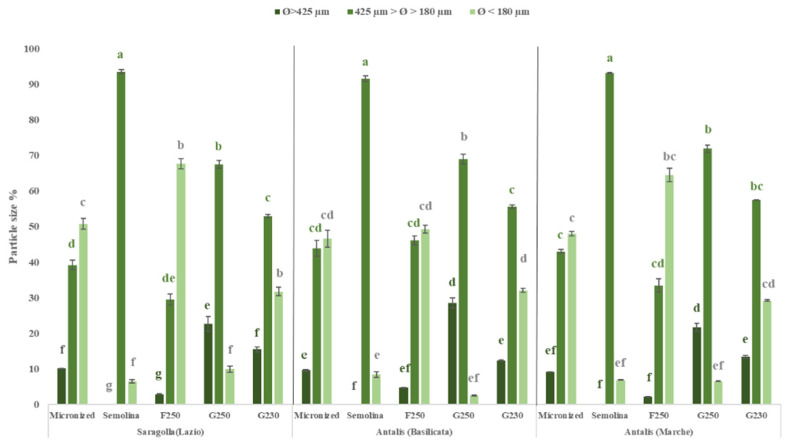
Mean particle size fractions (%) of micronized samples, semolina and air-classified fractions (F250, G250 and G230) obtained from Saragolla_LA, Antalis_BA and Antalis_MA. Different letters indicate statistically significant difference (*p* < 0.05, *n* = 2) within each cultivar sample.

About particle size (Ø), in semolina samples the range was between 425 and 180 µm, whereas in air-classified fractions varied depending on the type of fraction (G or F) (Figure 3).

F fractions had a higher concentration of fine particles (Ø < 180 µm), which was between 49% (Antalis_MA) and 68% (Saragolla_LA). By contrast, G fractions contained more coarse particles (Ø > 425 µm) with contents ranging from 12% to 28% (Antalis_BA). The content of intermediate particles and fine particles of all air-classified fractions (except for G250) was significantly different (*p* < 0.05) from that found in semolina samples (Figure 3).

### 3.3. Ash Content

**Figure 4:**
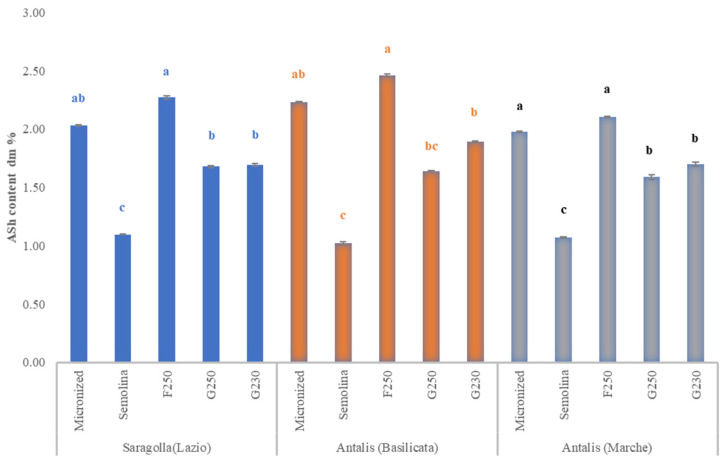
Mean values of ash content (d.m.%) of five milling products: micronized sample, semolina, F250, G250 and G230 air-classified fractions obtained from Saragolla_LA, Antalis_BA and Antalis_MA. Different letters indicate statistically significant differences (*p* < 0.05, *n* = 3) within each cultivar sample.

In general, ash content of micronized samples and bran-enriched fractions was significantly (*p* < 0.05) higher compared to that of semolina (Figure 4). Among F fractions, ash content ranged from 2.46 (Antalis_BA) to 2.10 (Antalis_MA), which was significantly higher (*p* < 0.05) than in G, varying from 1.89 (Antalis BA) to 1.59 (Antalis_MA).

### 3.4. Alveographic Properties of Air-Classified Fractions

Alveographic parameters of air-classified fractions, in comparison with the corresponding semolina samples, showed an increased P/L ratio (Table 2). While semolina samples showed P/L ratios ranging from a minimum of 1.49 ± 0.15 (Antalis_BA) to a maximum of 3.96 ± 0.38 (Saragolla_LA), higher values were observed in air-classified fractions, varying from 4.69 ± 0.21 (Antalis_BA G250) to 11.32 ± 0.08 (Antalis_MA F250). The W parameters showed more homogeneous values between air-classified fractions and semolina and a narrower range of variation (i.e., from 133 ± 20 J·10^−4^ (Antalis_BA F250) to 286 ± 48 J·10^−4^ (Antalis_MA semolina).

### 3.5. Starch Composition of Air-Classified Fractions

Starch, amylose and damaged starch contents were determined in the air-classified fractions (F250, G230 and G250) along with semolina and micronized samples (Table 3). Total starch content was significantly lower in micronized samples and in all air-classified samples as compared to semolina (*p* < 0.05). Saragolla_LA showed the highest amount of total starch (between 61.19% and 74.87%), followed by Antalis_BA (55.09–67.68%) and Antalis_MA (53.01–64.83%). G fractions had superior amounts of total starch than F fractions (Table 3). Interestingly, damaged starch values were not significantly different (*p* > 0.05) between F50 fraction and semolina, whereas significant differences (*p* < 0.05) resulted between each G fraction and the corresponding semolina sample.

The amylose content was directly determined on semolina, micronized samples and air-classified fractions. Results showed a significant reduction of amylose content in all air-classified fractions and micronized samples due to the reduced amount of starch as compared to semolina (Table 3). However, this reduction was not significant (*p* > 0.05) in the case of evaluation of the amylose/total starch ratio (data not shown).

### 3.6. Phenolic Acids Analysis

Phenolic acids content and composition of micronized samples, F250, G250 and G230 air classified fractions, and semolina from three grain samples are shown in Table 4. Overall, each milling product exhibited a typical phenolic acid profile, differing significantly (*p* < 0.05) for almost all individual components. Independently of the grain sample and milling type, ferulic acid was the most abundant phenolic acid being comprised between 63.52 µg/g dry matter (Antalis_BA, semolina) and 569.60 µg/g dry matter (Antalis_BA, F250). Second in abundancy was sinapic acid, ranging from 8.28 µg/g dry matter (Saragolla_LA, semolina) to 68.26 µg/g dry matter (Antalis_MA, F250).

Four minor compounds were also detected, namely sinapic, *p*-coumaric, vanillic, syringic and *p*-hydroxybenzoic acids (Table 4).

Semolina had the lowest concentration of all phenolic acids, whereas the micronized samples and the F250 fraction had the highest (Table 4).

**Figure 5:**
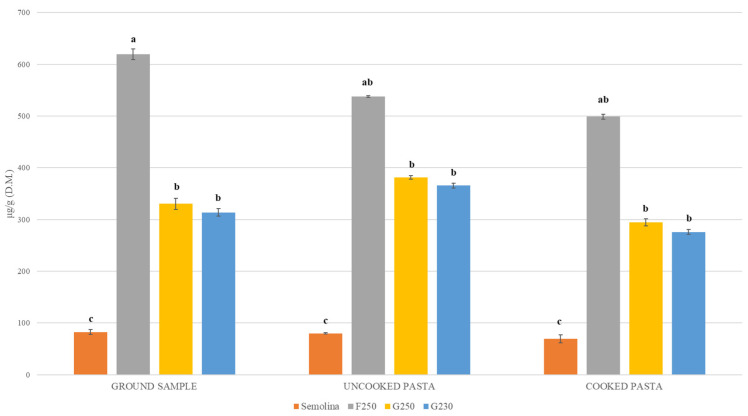
Average contents (*n* = 3) of total phenolic acids (TPAs), expressed as μg/g dry matter (d.m.) of four milling types: semolina, F250, G250 and G230 air classified fractions, obtained from the overall durum grain samples used in this study, and derived uncooked and cooked spaghetti. Different letters show significant differences (*p* < 0.05).

The antioxidant activity associated with phenolic acids was lower in semolina samples (ranging from 2.07 to 2.67 µeq Trolox/g dry matter) compared to that of all air-classified fractions; the highest value was in Saragolla_LA, for the F250 fraction (15.22 µeq Trolox/g dry matter) (Table 4). Considering the average values of TPAs evaluated on the overall data across cultivars and environments, significant differences (*p* < 0.05) were found among the different milling products (Figure 5). The observed variation ranged from a minimum of 82.48 µg/g dry matter (semolina) to a maximum of 619.57 µg/g dry matter (F250). Compared to semolina, the F250 fraction had the highest TPAs content (7.5-fold vs. semolina), whereas the G250 and G230 fractions showed a four-fold higher content. The pasta-making and cooking processes caused a slight decrease of TPAs content compared to that of raw materials (Figure 5). Nevertheless, uncooked pasta made with air-classified fractions had significant enrichments of TPAs compared to traditional pasta made with semolina; such an increase varied from seven-fold (F250) to four-fold (G250 and G230) (Figure 5). A complete and detailed view of the phenolic acids profiles and antioxidant activity of uncooked and cooked pasta is presented in Supplementary Tables S1 and S2.

### 3.7. ATIs Content

**Figure 6:**
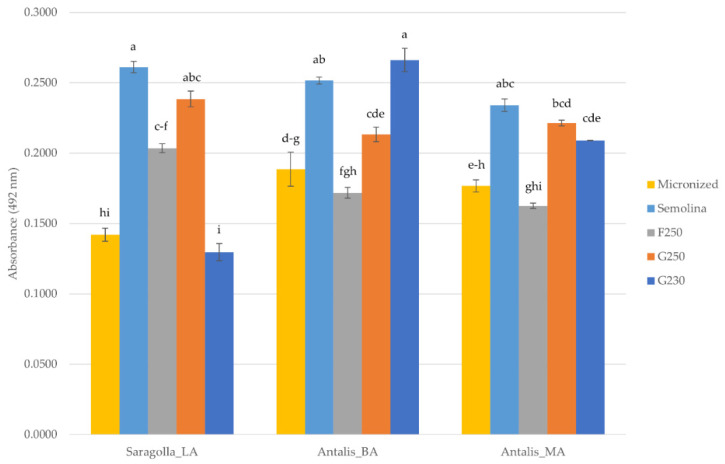
Indirect ELISA performed with anti-ATI polyclonal antibodies dilution 1:200 of micronized samples, semolina and air-classified fractions (F250, G250, G230) obtained from the durum grain samples used in this study. Different letters indicate significant differences (*p* < 0.05).

An ELISA test was performed for detecting differences in the amount of ATI on the albumin and globulin fractions (A/G) of the different milling types (micronized grains, F250, G250, and G230 air-classified fractions, and semolina). Data relative to the four antibody dilutions used are reported in Supplementary Table S3. Since the dilution 1:200 corresponded to the most linear region, it was used to build the histograms reported in Figure 6. Overall, results showed that semolina had higher ATIs contents compared to the other milling products, excepting for the Antalis_BA G230 air-classified fraction that had slightly higher, though not significant (*p* < 0.05), amounts (Figure 6). Among the five milling types obtained from the Saragolla_LA grain sample, the G230 fraction showed the lowest amount of ATI, whereas both Antalis_BA and Antalis_MA displayed the lowest content of ATI in the F250 air classified fraction (Figure 6).

## 4. Discussion

One of the key challenges to enhance the health value of durum wheat foods is the use of raw materials rich in health-promoting compounds and poor in toxic components. Bran is the compartment accounting for only 15% of the wheat grain, but it is an important source of components associated with health benefits, such as phenolic acids. To maintain these healthy bran components, and reduce the negative impacts associated with some endosperm proteins, such as ATIs, diverse bran-rich streams can be used [16,17].

In this paper, we evaluated several qualitative features of F250, G230 and G250 air-classified fractions as compared to semolina and micronized samples. The use of this updated technology directly provided several unrefined milling products, each having different characteristics, and without adding fractions rich in bioactive compounds [19]. This fact could lead to time and money savings, especially in a large-scale application.

First, milling yield percentages were assessed as they are among the major milling quality features [33]. The total recovery of milling products (F + G fractions) along the micronizing and air classification processes reached values around 99%, thus ensuring a minimal loss of raw material. The present results were comparable to those observed in previous evaluations [17]; slight differences detected here could depend on diverse grain size and texture [34]. The operating conditions that we used gave rise to G230 and F250 fractions with yield values that were near or above 60%, with some differences depending on grain samples (*p* < 0.05) [17].

The distribution of coarse, intermediate and fine particles within each G and F fraction resulted in agreement with a preliminary study [17]. The variability observed among the overall samples depended on grain samples and the setting valve point, supporting previous findings [17]. The typical particle size distribution characterizing each fraction type suggested that each could be more suitable to make specific unrefined end-products (pasta, bread, biscuits, etc.). The presence of bran particles in different rates within each air-classified fraction could lead to durum end-products (such as pasta) of higher quality than those obtained by adding bran aliquots to semolina [35]. The occurrence of coarse semolina particles in milling products could be important to enhance the gluten index and yellow color of end products [36]. Results regarding ash characterization suggested a higher content of minerals in the F fractions than in G fractions [37]. Overall, ash values were higher in air-classified fractions and micronized samples than in semolina. On the whole, results concerning yield, ash content and particle sizes’ composition proved to be in agreement with previous studies [17]. The alvegraphic behavior of the unrefined samples revealed a great reduction in the alveographic parameters, especially with regard to a P/L ratio increase. This latter observation confirms previous results on the quality effects of the addition of bran aliquots to semolina [38]. Total starch content was lower in micronized flour and in all air-classified fractions compared to semolina. G fractions had superior amounts of total starch compared to two out of three F250 samples. These data were expected, since F fractions contain a higher amount of bran and a reduced content of endosperm. Results of amylose content showed a significant reduction in all air-classified fractions and micronized samples compared to semolina due to the reduced amount of starch in these fractions. In this regard, when considering the amylose/total starch, no significant differences were detected. The reduction of starch, observed in the air-classified fraction compared to semolina, is potentially interesting for the realization of low glycemic foods, useful for the prevention of some diet-related diseases (type II diabetes, obesity and cardiovascular disorders) [13,14,15].

Based on phenolic acids analysis of raw materials and derived uncooked and cooked pasta, six main individual phenolic acids were identified, namely ferulic acid, vanillic acid, *p*-coumaric acid, sinapic acid, and syringic and *p*-hydroxybenzoic acids. These findings were in line with previous works carried out along the durum chain from seed to pasta [22,39]. Literature studies established that phenolic acids have important biological effects, independently if they are easily absorbed by the small intestine as free forms [40] or reach the colon intact as bound forms [41]. Upon these evidences, we estimated the overall content of free and bound forms for each individual phenolic acid [22,39]. Results showed that semolina had the lowest concentration of all individual phenolic components, whereas micronized samples and the F250 fraction had the highest (Table 4). That was expected for the diverse endosperm concentration in semolina and milling in which bran occurred, though at a different rate [38]. Considering the total sum of individual phenolic acids, the F250 fraction had the highest increase over semolina (650%) (Figure 5), suggesting that it would be the best to use for enriching the content of phenolic acids of pasta. In fact, the F250-derived pasta had the highest content of TPAs with an increase by up to 616% over that made with semolina (Figure 5). A slight, though not significant TPAs reduction (between 11% and 19%) resulted in cooked pasta compared to raw materials (Figure 5). These findings were expected and in line with other studies [39,42]. The antioxidant activity of phenolic extracts from raw materials and derived pasta were in good agreement with predictions considering the ferulic acid content with a TEAC of 3.0, indicating that the main source of antioxidant power of tested samples are the extracted phenolic acids.

Regarding ATIs, the highest amounts were found in semolina, which was expected since these components are mostly present in the starchy endosperm as reviewed [7]. So far, it can be concluded that our data show that micronization and air-classification treatment decrease the amount of ATIs, giving a potential added value to derived pasta products. The importance of ATIs in triggering adverse reactions to wheat is increasing, since they seem to be not only involved in allergies and sensitivity, but also in apparently different pathologies, such as Alzheimer’s disease, at least in murine models [43]. Thus, increasing attention is paid to the different procedures that can be used to achieve the goal of decreasing ATIs amounts. ATIs can be reduced in wheat flours through food processing, but the results are ambiguous (reviewed in [7]). Moreover, most of these processing procedures include fermentation, which is not typically used for pasta production. A genetic approach could theoretically be the method of choice; for example, transgenic and genome edited plants were produced in previous papers [44,45]. However, due to legislation restrictions, these genotypes cannot be used for commercial purposes. In addition, the production of new plant lines/varieties takes a long time. The air-fractionation procedure, described in this paper, has the advantage that it can be applied to different genotypes without the issues related to the above reported strategies, giving the possibility to produce pasta with high technological quality.

## 5. Conclusions

In the present work, F250, G230 and G250 air-classified fractions were characterized for standard quality parameters, starch, phenolic acids and ATIs content. The percentage distribution of coarse, intermediate and fine particles within each G and F fraction depended both on grain samples and setting valve points. While the rheological behavior revealed a reduction in the alveographic parameters, all fractions had significant improvements in other qualitative properties. In fact, total starch content diminished in micronized samples and in all air-classified fractions compared to semolina, suggesting their use for the production of low glycemic foods. All air-fractionated millings, especially F250, also showed strong improvements in phenolic acids content and antioxidant activity versus semolina and traditional pasta. Finally, micronization and air-classification treatments decreased the amount of ATIs. Overall, our data suggest the potential use of the F250, G230 and G250 air-classified fractions to make more nutritious, healthier and safer foods.

## Figures and Tables

**Table 1:** *Infratec*™ analysis: test weight, moisture, protein content, gluten and yellow color of durum wheat grain samples Saragolla_LA, Antalis_BA, Antalis_MA. Results indicate mean values of replicated analyses (*n* = 10); d.m. = dry matter.

Durum Wheat Grain Sample	Test Weight (kg/hl)	Moisture (%)	Protein Content (% d.m.)	Gluten (% d.m.)	Yellow Color Index
Saragolla_LA	83.4	10.2	12.7	8.7	14.1
Antalis_BA	80.2	10.2	13.9	9.8	15.0
Antalis_MA	81.7	10.3	15.0	10.5	13.6

**Table 2:** Alveographic parameters of semolina and F250, G250 and G230 air-classified fractions obtained from three durum grain samples (Saragolla_LA, Antalis_BA, Antalis_MA). Different letters indicate significant differences (*p* < 0.05) within each milling products; *n* = 5.

Durum Wheat Grain Samples	Milling Products	W(J·10^−4^)	P/L
**Saragolla_LA**	Semolina	241 ± 18 ^a^	3.96 ± 0.38 ^a^
**Antalis_BA**	Semolina	162 ± 7 ^b^	1.49 ± 0.15 ^b^
**Antalis_MA**	Semolina	286 ± 48 ^a^	3.85 ± 0.93 ^a^
**Saragolla_LA**	F250	129 ± 13 ^a^	10.80 ± 0.37 ^a^
**Antalis_BA**	F250	133 ± 20 ^a^	9.32 ± 0.66 ^b^
**Antalis_MA**	F250	156 ± 13 ^a^	11.32 ± 0.08 ^a^
**Saragolla_LA**	G250	144 ± 10 ^b^	8.35 ± 0.60 ^a^
**Antalis_BA**	G250	137 ± 5 ^b^	4.69 ± 0.21 ^c^
**Antalis_MA**	G250	189 ± 4 ^a^	7.50 ± 0.31 ^b^
**Saragolla_LA**	G230	144 ± 28 ^b^	8.93 ± 0.93 ^a^
**Antalis_BA**	G230	151 ± 6 ^b^	6.82 ± 0.58 ^b^
**Antalis_MA**	G230	217 ± 19 ^a^	7.59 ± 0.95 ^ab^

**Table 3:** Total starch, damaged starch and amylose (% d.m.) of five milling products: micronized sample, air-classified fractions (F250, G250, G230) and semolina obtained from three durum grain samples (Saragolla_LA, Antalis_BA, Antalis_MA). Different letters indicate statistically significant differences (*p* < 0.05, *n* = 3) within each milling product (*n* = 4).

Durum Wheat Grain Sample	Milling Product	Total Starch	Damaged Starch	Amylose
Saragolla_LA	Micronized	61.82 ± 0.94 ^cd^	4.88 ± 0.21 ^d^	15.23 ± 2.99 ^hi^
	Semolina	74.87 ± 1.45 ^a^	5.96 ± 0.56 ^abc^	26.70 ± 3.41 ^ab^
	F250	61.19 ± 1.23 ^d^	6.41 ± 0.68 ^ab^	14.02 ± 1.17 ^i^
	G250	67.49 ± 1.20 ^b^	2.62 ± 0.27 ^e^	21.56 ± 2.29 ^defg^
	G230	65.18 ± 1.44 ^b^	2.93 ± 0.11 ^e^	17.41 ± 1.02 ^ghi^
Antalis_BA	Micronized	56.46 ± 1.11 ^fg^	5.32 ± 0.39 ^cd^	21.67 ± 3.82 ^def^
	Semolina	67.68 ± 1.61 ^b^	5.80 ± 0.48 ^bcd^	28.84 ± 2.18 ^a^
	F250	55.09 ± 1.07 ^gh^	5.91 ± 0.22 ^abc^	18.39 ± 2.05 ^fgh^
	G250	60.26 ± 1.78 ^de^	2.76 ± 0.13 ^e^	23.80 ± 0.76 ^bcde^
	G230	59.26 ± 1.58 ^def^	3.51 ± 0.10 ^e^	25.54 ± 4.11 ^abcd^
Antalis_MA	Micronized	54.70 ± 1.36 ^gh^	5.38 ± 0.54 ^cd^	20.30 ± 1.58 ^efg^
	Semolina	64.83 ± 0.93 ^bc^	6.36 ± 0.29 ^ab^	26.09 ± 1.42 ^abc^
	F250	55.14 ± 1.25 ^gh^	6.83 ± 0.43 ^a^	18.82 ± 1.31 ^fgh^
	G250	53.01 ± 1.01 ^h^	3.02 ± 0.27 ^e^	23.75 ± 3.05 ^bcde^
	G230	57.13 ± 0.67 ^efg^	3.29 ± 0.08 ^e^	22.46 ± 2.43 ^cdef^

**Table 4:** Phenolic acid profiles (μg/g dry matter) and antioxidant activity (µeq Trolox/g dry matter) of micronized samples, air-classified fractions (F250, G250, G230) and semolina obtained from three durum grain samples (Saragolla_LA, Antalis_BA, Antalis_MA). Different letters within columns indicate significant differences (*p* < 0.05).

Durum Grain Sample	Milling Product	*p*-Hydroxy Benzoic Acid	Syringic Acid	Vanillic Acid	*p*-Coumaric Acid	Ferulic Acid	Sinapic Acid	TEAC
Saragolla_LA	Micronized	4.37 ± 0.50 ^ab^	5.98 ± 0.21 ^abc^	8.51 ± 0.48 ^bc^	7.96 ± 0.52 ^cd^	502.19 ± 8.05 ^b^	51.04 ± 1.64 ^abc^	8.10 ± 1.54 ^cde^
	Semolina	1.12 ± 0.01 ^cd^	1.43 ± 0.04 ^fg^	1.50 ± 0.42 ^f^	0.14 ± 0.01 ^f^	80.32 ± 2.79 ^e^	8.28 ± 0.66 ^g^	2.67 ± 0.18 ^gh^
	F250	6.22 ± 0.36 ^a^	8.12 ± 0.4 ^a^	11.83 ± 0.35 ^a^	11.60 ± 0.73 ^ab^	498.04 ± 5.08 ^b^	61.72 ± 3.83 ^ab^	15.22 ± 0.27 ^a^
	G250	2.96 ± 0.47 ^bcd^	3.23 ± 0.57 ^defg^	5.07 ± 0.83 ^de^	3.52 ± 0.62 ^ef^	383.78 ± 7.31 ^c^	32.38 ± 4.01 ^cde^	7.05 ± 0.58 ^cdef^
	G230	3.07 ± 0.44 ^bcd^	4.12 ± 0.51 ^cde^	5.59 ± 0.65 ^cd^	4.33 ± 0.47 ^e^	408.18 ± 10.25 ^c^	30.50 ± 3.49 ^cdef^	8.58 ± 0.38 ^bcd^
Antalis_BA	Micronized	3.36 ± 0.75 ^bc^	5.12 ± 0.19 ^bcd^	5.92 ± 0.26 ^cd^	8.59 ± 0.11 ^bc^	409.65 ± 4.24 ^c^	41.47 ± 4.12 ^bcde^	9.45 ± 0.05 ^bc^
	Semolina	0.76 ± 0.15 ^d^	0.76 ± 0.18 ^g^	0.73 ± 0.09 ^f^	0.09 ± 0.00 ^f^	63.52 ± 3.32 ^e^	9.81 ± 0.47 ^fg^	2.47 ± 0.06 ^gh^
	F250	4.72 ± 0.56 ^ab^	8.15 ± 0.20 ^a^	7.53 ± 0.62 ^bc^	13.02 ± 0.59 ^a^	569.60 ± 5.41 ^a^	44.21 ± 4.77 ^bcd^	12.26 ± 0.17 ^ab^
	G250	2.07 ± 0.08 ^bcd^	1.60 ± 0.75 ^fg^	2.12 ± 0.70 ^f^	2.17 ± 1.00 ^ef^	240.90 ± 6.59 ^d^	25.36 ± 1.38 ^defg^	5.03 ± 0.15 ^efgh^
	G230	1.53 ± 0.12 ^cd^	1.64 ± 0.23 ^efg^	2.22 ± 0.28 ^f^	2.81 ± 0.74 ^ef^	203.66 ± 5.87 ^d^	20.59 ± 2.11 ^efg^	5.48 ± 0.17 ^defg^
Antalis_MA	Micronized	3.12 ± 0.01 ^bc^	4.69 ± 0.33 ^cd^	6.76 ± 0.15 ^bcd^	8.49 ± 0.30 ^bc^	394.44 ± 4.44 ^c^	54.71 ± 5.03 ^ab^	9.07 ± 0.39 ^bc^
	Semolina	0.83 ± 0.01 ^cd^	1.01 ± 0.01 ^fg^	1.19 ± 0.11 ^f^	0.16 ± 0.00 ^f^	67.51 ± 3.48 ^e^	10.99 ± 0.05 ^fg^	2.07 ± 0.10 ^h^
	F250	4.04 ± 0.11 ^ab^	7.73 ± 0.58 ^ab^	9.09 ± 0.49 ^ab^	12.40 ± 0.40 ^a^	512.48 ± 4.37 ^b^	68.26 ± 3.61 ^a^	11.90 ± 0.67 ^ab^
	G250	1.91 ± 0.01 ^bcd^	4.49 ± 0.53 ^cd^	3.42 ± 0.03 ^def^	4.57 ± 0.21 ^de^	241.48 ± 5.66 ^d^	29.74 ± 2.59 ^def^	4.26 ± 0.02 ^fgh^
	G230	1.88 ± 0.01 ^cd^	3.49 ± 0.04 ^cdef^	3.18 ± 0.27 ^def^	4.28 ± 0.35 ^e^	214.11 ± 4.38 ^d^	25.47 ± 1.62 ^defg^	4.32 ± 0.17 ^fgh^

## Data Availability

The data is contained within the article.

## Supplementary Materials

The following are available online at https://www.mdpi.com/article/10.3390/foods10112817/s1, Table S1: Phenolic acid profiles (μg/g dry matter) and antioxidant activity (µeq Trolox/g dry mat-ter) of un-cooked pasta made with air-classified fractions (F250, G250, G230) and semolina ob-tained from three durum grain samples (Saragolla_LA, Antalis_BA, Antalis_MA), Table S2: Phe-nolic acid profiles (μg/g dry matter) and antioxidant activity (µeq Trolox/g dry matter) of cooked pasta made with air-classified fractions (F250, G250, G230) and semolina obtained from three durum grain samples (Saragolla_LA, Antalis_BA, Antalis_MA), Table S3: Anti-ATI polyclonal antibodies on micronized wholemeal, F250, G250, G230 air-classified fractions, and semolina from three durum grain samples.

## References

[1] Hemery Y., Rouau X., Lullien-Pellerin V., Barron J.C., Abecassis J. (2007). Dry processes to develop wheat fractions and products with enhanced nutritional quality. J. Cereal Sci..

[2] Sevgi K., Tepe B., Sarikurkcu C. (2015). Antioxidant and DNA damage protection potentials of selected phenolic acids. Food Chem. Toxicol..

[3] Pandey K.B., Rizvi S.I. (2009). Plant polyphenols as dietary antioxidants in human health and disease. Oxidative Med. Cell. Longev..

[4] Calabriso N., Massaro M., Scoditti E., Pasqualone A., Laddomada B., Carluccio M.A. (2020). Phenolic extracts from whole wheat biofortified bread dampen overwhelming 1 inflammatory response in human endothelial cells and monocytes: Major role of VCAM-1 and CXCL-10. Eur. J. Nutr..

[5] Laddomada B., Blanco A., Mita G., D’Amico LSingh R.P., Ammar K., Crossa J., Guzmán C. (2021). Drought and heat stress impacts on phenolic acids accumulation in durum wheat cultivars. Foods.

[6] Nigro D., Grausgruberb H., Guzmàn C., Laddomada B., Igrejas G., Ikeda T., Guzmàn C. (2020). Phenolic compounds in wheat kernels: Genetic and genomic studies of biosynthesis and regulation. Wheat Quality for Improving Processing and Health.

[7] Geisslitz S., Shewry P., Brouns F., America A.H.P., Caio G.P.I., Daly M., D’Amico S., De Giorgio R., Gilissen L., Grausgruber H. (2021). Wheat ATIs: Characteristics and Role in Human Disease. Front. Nutr..

[8] Otles S., Ozgoz S. (2014). Health effects of dietary fiber. Acta Sci. Pol. Technol. Aliment..

[9] Probst Y.C., Guan V.X., Kent K. (2017). Dietary phytochemical intake from foods and health outcomes: A systematic review protocol and preliminary scoping. Br. Med. J..

[10] Stephen A.M., Champ M.M.J., Cloran S.J., Fleith M., van Lieshout L., Mejborn H., Burley V.J. (2017). Dietary fibre in Europe: Current state of knowledge on definitions, sources, recommendations, intakes and relationships to health. Nutr. Res. Rev..

[11] Jacobs D., Anderson F.L., Blomhoff R. (2007). Wholegrain consumption is associated with a reduced risk of non cardiovascular, non cancer death attributed to inflammatory disease in the Iowa Women’s Health Study. Am. J. Clin. Nutr..

[12] Kushi L.H., Meyer K.A., Jacobs D.R. (1999). Cereals, legumes, and chronic disease risk reduction: Evidence from epidemiologic studies. Am. J. Clin. Nutr..

[13] Aune D., Keum N.N., Giovannucci E., Fadnes L.T., Boffetta P., Greenwood D.C., Tonstad S., Vatten L.J., Riboli E., Norat T. (2016). Whole grain consumption and risk of cardiovascular disease, cancer, and all cause and cause specific mortality: Systematic review and dose-response meta-analysis of prospective studies. Br. Med. J..

[14] Călinoiu L.F., Vodnar D.C. (2018). Whole grains and phenolic acids: A review on bioactivity, functionality, health benefits and bioavailability. Nutrients.

[15] Giacco R., Costabile G., Della Pepa G., Anniballi G., Griffo E., Mangione A., Cipriano P., Viscovo D., Clemente G., Landberg R. (2014). A whole-grain cereal-based diet lowers postprandial plasma insulin and triglyceride levels in individuals with metabolic syndrome. Nutr. Metab. Cardiovasc. Dis..

[16] Hemery Y., Chaurand M., Holopainen U., Lampi A.-M., Lehtinen P., Piironen V., Sadoudi A., Rouau X. (2011). Potential of dry fractionation of wheat bran for the development of food ingredients, part I: Influence of ultra-fine grinding. J. Cereal Sci..

[17] Cammerata A., Sestili F., Laddomada B., Aureli G. (2021). Bran-Enriched Milled Durum Wheat Fractions Obtained Using Innovative Micronization and Air-Classification Pilot Plants. Foods.

[18] Cammerata A., Marabottini R., Allevato E., Aureli G., Stazi S.R. (2021). Content of minerals and deoxynivalenol in the air-classified fractions of durum wheat. Cereal Chem. J..

[19] Ficco D.B.M., Borrelli G.M., Giovanniello V., Platani C., De Vita P. (2018). Production of anthocyanin-enriched flours of durum and soft pigmented wheats by air-classification, as a potential ingredient for functional bread. J. Cereal Sci..

[20] Gomez-Caravaca A.M., Verardo V., Candigliota T., Marconi E., Segura-Carretero A., Fernandez-Gutierrez A., Caboni M.F. (2015). Use of air classification technology as green process to produce functional barley flours naturally enriched of alkylresorcinols, β-glucans and phenolic compounds. Food Res. Int..

[21] Laudadio V., Bastoni E., Introna M., Tufarelli V. (2013). Production of low-fiber sunflower (*Helianthus annuus* L.) meal by micronization and air classification processes. CyTA-J. Food.

[22] Chen Z., Li W., Ren W., Li Y., Chen Z. (2014). Triboelectric separation of aleurone cell-cluster from wheat bran fragments in nonuniform electric field. Food Res. Int..

[23] Chen Z., Zha B., Wang L., Wang R., Chen Z., Tian Y. (2013). Dissociation of aleurone cell cluster from wheat bran by centrifugal impact milling. Food Res. Int..

[24] EN ISO 2171:2010 (2010). Cereals, Pulses and by-Products-Determination of Ash Yield by Incineration (ISO 2171:2007).

[25] (2001). Decreto del Presidente della Repubblica 9 febbraio 2001, n. 187. Regolamento per la Revisione Della Normativa Sulla Produzione e Commercializzazione di Sfarinati e Paste Alimentari, a Norma Dell’articolo 50 Della Legge 22 feb-Braio 1994, n. 146.

[26] (1995). AACC Method 54-30.02 and UNI 10453. Alveograph Method for Soft and Hard Wheat Flour.

[27] Chrastil J. (1987). Improved colorimetric determination of amylose in starches or flours. Carbohydr. Res..

[28] Laddomada B., Durante M., Mangini G., D’Amico L., Lenucci M.S., Simeone R., Piarulli L., Mita G., Blanco A. (2017). Genetic variation for phenolic acids concentration and composition in a tetraploid wheat (*Triticum turgidum* L.) collection. Genet. Resour. Crop. Evol..

[29] Durante M., Milano F., Caroli M., Giotta L., Piro G., Mita G., Frigione M., Lenucci M.S. (2020). Tomato Oil Encapsulation by α-, β-, and γ-Cyclodextrins: A Comparative Study on the Formation of Supramolecular Structures, Antioxidant Activity, and Carotenoid Stability. Foods.

[30] Lupi R., Denery-Papini S., Rogniaux H., Lafiandra D., Rizzi C., De Carli M., Moneret-Vautrin D.A., Masci S., Larré C. (2013). How much does transgenesis affect wheat allergenicity?: Assessment in two GM lines over-expressing endogenous genes. J. Proteom..

[31] Hammer O., Harper D.A.T., Ryan P.D. (2001). PAST: Paleontological Statistic Software Package for Education and Data Analysis. Paleontol. Electron..

[32] UNI 10709:1998 (1998). Durum Wheat Grains. Qualitative Requirements, Classification and Test Methods.

[33] Sabanci K., Aydin N., Sayaslan A., Sonmez M.E., Aslan M.F., Demir L., Sermet C. (2020). Wheat Flour Milling Yield Estimation Based on Wheat Kernel Physical Properties Using Artificial Neural Networks. Int. J. Intell. Syst. Appl. Eng..

[34] Marshall D.R., Mares D.J., Moss H.I., Ellison F.W. (1986). Effects of grain shape and size on milling yields in wheat. II. Experimental studies. Aust. J. Agric. Res..

[35] Alzuwaid N.T., Fellows C.M., Laddomada B., Sissons M. (2020). Impact of wheat bran particle size on the technological and phytochemical properties of durum wheat pasta. J. Cereal Sci..

[36] Sacchetti G., Cocco G., Cocco D., Neri L., Mastrocola D. (2011). Effect of semolina particle size on the cooking kinetics and quality of spaghetti. Procedia Food Sci..

[37] Padalino L., Mastromatteo M., Lecce L., Spinelli S., Conte A., Del Nobile M.A. (2015). Effect of raw material on cooking quality and nutritional composition of durum wheat spaghetti. Int. J. Food Sci. Nutr..

[38] Pasqualone A., Laddomada B., Centomani I., Paradiso V.M., Minervini D., Caponio F., Summo C. (2017). Bread making aptitude of mixtures of re-milled semolina and selected durum wheat milling by-products. LWT-Food Sci. Technol..

[39] Martini D., Ciccoritti R., Nicoletti I., Nocente F., Corradini D., D’Egidio M.G., Taddei F. (2017). From seed to cooked pasta: Influence of traditional and non-conventional transformation processes on total antioxidant capacity and phenolic acid content. Int. J. Food Sci. Nutr..

[40] Manach C., Scalbert A., Morand C., Rémésy C., Jiménez L. (2004). Polyphenols: Food sources and bioavailability. Am. J. Clin. Nutr..

[41] Cardona F., Andres-Lacueva C., Tulipani S., Tinahones F.J., Queipo-Ortuno I.M. (2013). Benefits of polyphenols on gut microbiota and implications in human health. J. Nutr. Biochem..

[42] Fares C., Platani C., Baiano A., Menga V. (2010). Effect of processing and cooking on phenolic acid profile and antioxidant capacity of durum wheat pasta enriched with debranning fractions of wheat. Food Chem..

[43] dos Santos Guilherme M., Zevallos V.F., Pesi A., Stoye N.M., Nguyen V.T.T., Radyushkin K., Schwiertz A., Schmitt U., Schuppan D., Endres K. (2020). Dietary Wheat Amylase Trypsin Inhibitors Impact Alzheimer’s Disease Pathology in 5xFAD Model Mice. Int. J. Mol. Sci..

[44] Kalunke R.M., Tundo S., Sestili F., Camerlengo F., Lafiandra D., Lupi R., Larrè C., Denery-Papini S., Islam S., Ma W. (2020). Reduction of allergenic potential in bread wheat RNAi transgenic lines silenced for CM3, CM16 and 0.28 ATI genes. Int. J. Mol. Sci..

[45] Camerlengo F., Frittelli A., Sparks C., Doherty A., Martignago D., Larré C., Lupi R., Sestili F., Masci S. (2020). CRISPR-Cas9 multiplex editing of the α-amylase/trypsin inhibitor genes to reduce allergen proteins in durum wheat. Front Sustain. Food Syst..

