# Supplementary material for: Qualitative Characterization of Unrefined Durum Wheat Air-Classified Fractions
Source: Foods. 2021 Nov 16;10(11):2817. doi: 10.3390/foods10112817 (PMC8618629; doi:10.3390/foods10112817)
Supplement: Supplementary file 1 [file foods-10-02817-s001.zip › foods-1404574-supplementary.pdf]

Table S1. Phenolic acid profiles (μg/g dry matter) and antioxidant activity (μeq Trolox/g dry matter) of un-cooked pasta made with air-classified fractions (F250, G250, G230) and semolina obtained from three durum grain samples (Saragolla_LA, Antalis_BA, Antalis_MA). Same letters within columns indicate not significant difference ($p < 0.05$).

Grain sample	Milling product	*p*-Hydroxy benzoic acid	Syringic acid	Vanillic acid	*p*-Coumaric acid	Ferulic acid	Sinapic acid	TEAC
Saragolla_LA	F250	5.89±0.03^{a}	9.84±0.02^{a}	12.94±0.08^{a}	10.53±0.14^{a}	540.75±1.41^{a}	33.17±0.76^{a}	10.80±0.14^{a}
	G250	3.64±0.03^{b}	5.64±0.04^{b}	7.17±0.06^{bc}	4.87±0.08^{cd}	443.80±1.92^{c}	24.03±1.88^{b}	7.87±0.06^{b}
	G230	3.53±0.03^{b}	5.41±0.17^{b}	7.27±0.06^{bc}	4.92±0.06^{cd}	430.65±5.00^{c}	26.05±0.59^{b}	5.35±0.17^{c}
	Semolina	1.24±0.01^{cd}	1.88±0.01^{e}	1.22±0.01^{d}	0.07±0.01^{f}	70.20±2.23^{h}	3.32±0.03^{c}	1.14±0.03^{e}
Antalis_BA	F250	2.41±0.54^{bcd}	1.51±0.05^{e}	9.94±0.04^{ab}	8.38±0.14^{b}	506.15±4.86^{b}	23.36±0.52^{b}	8.52±0.12^{b}
	G250	2.57±0.01^{bc}	2.57±0.35^{de}	4.59±0.45^{cd}	5.48±0.02^{cd}	310.47±5.02^{ef}	28.15±1.27^{ab}	5.23±0.00^{c}
	G230	1.24±0.33^{cd}	1.51±0.06^{e}	2.39±0.12^{d}	2.39±0.56e	287.06±4.48^{f}	11.55±0.59^{c}	5.15±0.07^{c}
	Semolina	1.09±0.04^{d}	1.56±0.22^{e}	1.34±0.08^{d}	0.74±0.05^{ef}	69.25±0.05^{h}	6.63±0.35^{c}	1.11±0.01^{e}
Antalis_MA	F250	3.53±0.02^{b}	4.18±0.00^{bcd}	6.69±1.69^{bc}	6.73±0.11^{bc}	397.31±2.62^{d}	30.30±0.78^{ab}	6.24±0.26^{c}
	G250	3.36±0.21^{b}	4.92±0.64^{bc}	6.05±0.15^{c}	7.23±0.62^{bc}	260.09±4.64^{g}	18.49±0.33^{b}	3.80±0.43^{d}
	G230	2.73±0.12^{b}	3.57±0.12^{cd}	4.29±0.35^{cd}	4.73±0.16^{d}	273.84±3.36^{fg}	23.21±1.20^{b}	5.35±0.17^{c}
	Semolina	1.04±0.23^{d}	1.63±0.24^{e}	1.18±0.23^{d}	0.69±0.32^{ef}	71.77±1.40^{h}	6.33±0.33^{c}	1.11±0.01^{e}

Table S2. Phenolic acid profiles (μg/g dry matter) and antioxidant activity (μeq Trolox/g dry matter) of cooked pasta made with air-classified fractions (F250, G250, G230) and semolina obtained from three durum grain samples (Saragolla_LA, Antalis_BA, Antalis_MA). Same letters within columns indicate not significant difference ($p < 0.05$).

Grain sample	Milling product	*p*-Hydroxy benzoic acid	Syringic acid	Vanillic acid	*p*-Coumaric acid	Ferulic acid	Sinapic acid	TEAC
Saragolla_LA	F250	4.35±0.23 ^{a}	7.72±0.24^{a}	8.58±0.61^{a}	9.05±0.^{47a}	504.66±8.03^{a}	31.28±0.89^{a}	8.33±0.02^{a}
	G250	2.14±0.02^{bc}	3.38±0.04^{bc}	4.12±0.01^{bc}	3.13±0.01^{bcd}	340.66±7.48^{d}	24.55±0.07^{abc}	5.71±0.14^{b}
	G230	1.06±0.13^{cd}	1.23±0.04^{c}	1.68±0.21bc	1.39±0.66^{cde}	282.30±3.99^{e}	12.75±2.76^{ef}	5.17±0.05^{bc}
	Semolina	0.43±0.05^{d}	0.76±0.23^{c}	0.75±0.16^{c}	0.24±0.07^{e}	45.53±7.85^{h}	6.55±1.59^{f}	0.90±0.09^{f}
Antalis_BA	F250	1.73±0.03^{bc}	2.58±0.30^{bc}	4.08±0.93^{bc}	5.02±0.67^{b}	460.15±2.38^{b}	19.00±0.08^{bcde}	7.62±0.12^{a}
	G250	1.56±0.16^{bcd}	1.47±0.16^{bc}	2.36±0.45^{bc}	2.76±0.15^{bcde}	164.07±6.21^{g}	16.15±1.76^{de}	2.88±0.03^{e}
	G230	1.79±0.12^{bc}	2.16±0.16^{bc}	2.77±0.18^{bc}	3.92±0.37^{bc}	212.07±6.41^{f}	19.76±0.11^{bcde}	4.74±0.12^{cd}
	Semolina	0.80±0.13^{d}	0.95±0.10^{c}	0.83±0.12^{c}	0.43±0.28^{de}	57.26±8.06^{h}	6.95±1.01^{f}	0.90±0.00^{f}
Antalis_MA	F250	2.64±0.44^{b}	4.06±1.32^{b}	4.77±1.49^{ab}	8.33±0.54^{a}	402.29±5.75^{c}	16.71±0.43^{cde}	1.11±0.03^{f}
	G250	2.66±0.02^{b}	2.93±0.01^{bc}	3.44±0.04^{bc}	4.46±0.45^{b}	277.95±4.38^{e}	26.08±0.17^{ab}	5.19±0.15^{bc}
	G230	2.09±0.13^{bc}	2.36±0.08^{bc}	2.70±0.15^{bc}	3.47±0.06^{bcd}	252.14±5.37^{e}	21.76±1.28^{bcd}	4.74±0.01^{cd}
	Semolina	1.25±0.01^{cd}	1.56±0.04^{bc}	1.05±0.03^{bc}	0.83±0.03^{de}	73.25±3.69^{h}	9.57±0.12^{ef}	4.41±0.01^{d}

Table S3. Anti-ATI polyclonal antibodies on micronized wholemeal, F250, G250, G230 air-classified fractions, and semolina from three durum grain samples. Different letters indicate significant differences ($p<0.05$) within columns.

Durum wheat grain sample	Milling type	Dilution			
		1:20	1:200	1:2000	1:20000
Saragolla_LA	Micronized	0.25 ± 0.03^{efg}	0.14 ± 0.01^{hi}	0.08 ± 0.01^{g}	0.06 ± 0.00^{a}
	Semolina	0.36 ± 0.02^{ab}	0.26 ± 0.01^{a}	0.17 ± 0.01^{a}	0.08 ± 0.00^{a}
	F250	0.31 ± 0.01^{bcd}	$0.20\pm0.01^{c-f}$	0.11 ± 0.01^{def}	0.06 ± 0.00^{a}
	G250	0.33 ± 0.01^{bc}	0.24 ± 0.01^{abc}	0.14 ± 0.01^{bc}	0.08 ± 0.00^{a}
	G230	0.19 ± 0.00^{hi}	0.13 ± 0.01^{i}	0.14 ± 0.01^{bc}	0.09 ± 0.02^{a}
Antalis_BA	Micronized	0.30 ± 0.01^{cde}	$0.19\pm0.02^{d-g}$	0.10 ± 0.01^{ef}	0.06 ± 0.00^{a}
	Semolina	0.38 ± 0.00^{a}	0.25 ± 0.00^{ab}	0.15 ± 0.00^{b}	0.08 ± 0.01^{a}
	F250	$0.27\pm0.01^{d-g}$	0.17 ± 0.01^{fgh}	0.11 ± 0.01^{ef}	0.09 ± 0.02^{a}
	G250	0.33 ± 0.02^{bc}	0.21 ± 0.01^{cde}	0.12 ± 0.01^{cde}	0.08 ± 0.00^{a}
	G230	0.33 ± 0.00^{abc}	0.27 ± 0.01^{a}	0.15 ± 0.00^{ab}	0.09 ± 0.04^{a}
Antalis_MA	Micronized	0.22 ± 0.00^{gh}	$0.18\pm0.01^{e-h}$	0.11 ± 0.01^{ef}	0.06 ± 0.00^{a}
	Semolina	0.31 ± 0.00^{cd}	0.23 ± 0.01^{abc}	0.14 ± 0.00^{b}	0.08 ± 0.00^{a}
	F250	0.24 ± 0.01^{fg}	0.16 ± 0.00^{ghi}	0.10 ± 0.00^{fg}	0.06 ± 0.00^{a}
	G250	0.27 ± 0.01^{def}	0.22 ± 0.00^{bcd}	0.13 ± 0.00^{bcd}	0.07 ± 0.00^{a}
	G230	0.15 ± 0.01^{i}	0.21 ± 0.00^{cde}	0.13 ± 0.01^{bcd}	0.08 ± 0.01^{a}
